# Supplementary material for: Defining the transition from new to normal: a qualitative investigation of the clinical change process
Source: BMC Health Serv Res. 2024 Dec 18;24:1592. doi: 10.1186/s12913-024-12034-4 (PMC11653967; doi:10.1186/s12913-024-12034-4)
Supplement: Supplementary file 1 — Supplementary Material 1. [file 12913_2024_12034_MOESM1_ESM.docx]

**Codebook: Culture Change Project**

**Research question: What constitutes true “culture change” in clinical practice, meaning how do new evidence-based practices, guidelines, or approaches move beyond initial uptake and later sustainment, and then become the “new normal”?**

| **Domain** | **Code** | **Sub-code** | **Interview Question** |
| --- | --- | --- | --- |
| **Definition:** The perceived factors that define culture change in healthcare practice.  *Use these codes throughout the transcript as applicable, not only when the participant is asked about the definition of culture change. | **Time:** The amount of time required for a new practice to become a culture change. |  | What does culture change in health care practice mean to you? |
|  | **Magnitude:** The magnitude of the practice change (i.e., the extent to which it deviates from the status quo). |  |  |
|  | **Scale:** The scale of the practice change (i.e., change across a unit vs. organization vs. network). |  |  |
|  | **Integration:** The degree to which the practice is integrated into existing workflows. |  |  |
|  | **Sustainability:** The perceived relationships between sustainability and culture change. | **Connections:** The perceived connections between sustainability and culture change.  **Differences:** The perceived differences between sustainability and culture change.   1. **Staff buy-in:** Buy-in from staff regarding the value of the culture change. 2. **Normative restructuring:** True change in norms, values, and beliefs [or lack thereof] that govern action. 3. **Relational restructuring:** Change in the ways people are organized and relate to each other. 4. **Oversight:** Requirement [or lack thereof] of mandates and reminders to sustain change. | What do you see as the difference(s) between sustainability and culture change? |
| **Contextual factors:** the environmental stakeholders, structures, and events that affect the integration and sustainment of culture chance.  *Double code to Integration and Sustainment to indicate which is influenced.  *What contextual factors affect culture change?* | **Strategic intentions:** How the context affects the formulation and planning of culture change and its components. | **Available resources:** The level of resources dedicated for implementation of the change and on-going operations, including money, training, education, physical space, and time.  **Access to knowledge and information:** Ease of access to digestible information and knowledge about the change and how to incorporate it into work tasks.  Note that this code is to be used more broadly than “education” to capture the availability of information about the change, whether that be evidence and literature to gain staff buy-in and/or guidance on how to implement the change. | Please describe the environment in which it was implemented.  Why do you think this new practice resulted in culture change and not only time-limited change (or vice-versa)? |
|  | **Negotiating capacity:** How the context affects the extent that culture change can fit, or be integrated, into existing ways of working by the stakeholders. | **Tension for change:** The degree to which stakeholders perceive the current situation as intolerable or needing change.   1. **Evidence base:** Mismatch between current practices and the most updated and relevant evidence-based practices on delivering best quality of care.      1. **Peer pressure:** Mimetic or competitive pressure to implement an intervention; typically because most or other key peer or competing organizations have already implemented or are in a bid for a competitive edge. 2. **Policies and guidelines:** Strategies to spread interventions, including policy and regulations (governmental or other central entity), mandates, recommendations and guidelines, pay-for-performance, collaboratives, and public or benchmark reporting. 3. **Patient needs:** The knowledge and prioritization of patient needs to deliver the best quality of care.   **Perceived value:** Includes 1) individuals’ perception of the importance of the implementation within the organization and how the culture change aligns with individuals’ own norms, values, and perceived risks and needs and 2) overall organizational support and prioritization of the culture change.  **Compatibility:** The degree of tangible fit between the practice change and existing workflows and systems within the organization. | Please describe the environment in which it was implemented.  Why do you think this new practice resulted in culture change and not only time-limited change (or vice-versa)?  How did the idea for the new practice came about?  Why did the change in practice happen? |
|  | **Reframing organizational logistics:** How existing social structural and social cognitive resources shape the implementation environment for culture change to occur. | **Leadership engagement:** Support, involvement, and accountability of leaders and managers with the culture change.    **Staff engagement:** Commitment, involvement, and accountability of hospital staff with the culture change.  **Networks and communications:** The nature and quality of webs of social networks and the nature and quality of formal and informal communications within an organization. | Please describe the environment in which it was implemented.  Why do you think this new practice resulted in culture change and not only time-limited change (or vice-versa)? |
| **Mechanisms:** The social processes, approaches, and collaborative work that involve the investment of personal and group resources to achieve culture change.  *How is culture change enacted? What is the work that stakeholders do to create culture change?* | **Planning:** The degree to which methods and tasks for implementing an intervention are developed in advance, and the quality of those schemes or methods. |  | How did the idea for the new practice came about?  Why did the change in practice happen? |
|  | **Engaging:** Attracting and involving appropriate individuals in the implementation and use of the intervention through a variety of strategies. | 1. **Stakeholder:** The type of stakeholder who is being engaged in the culture change.   **Opinion leaders:** Individuals in an organization who have formal or informal influence on the attitudes and beliefs of their colleagues with respect to implementing the intervention.  **Champions:** Implementation leaders who support, market, and drive the practice change, overcoming indifference or resistance that the intervention may provoke.  **Workers:** The frontline workers who are engaging with the healthcare practice on a daily basis. | Who were the implementers?  Who were the stakeholders? |
|  |  | 1. **Engagement strategy:** The strategy used to engage stakeholders in the culture change.   **Social marketing:** Providing promotional materials about the practice change via social media platforms and in-person resources (e.g., paper flyers, posters, presentations, etc.).  **Education:** Providing educational resources and training materials on the practice change.  **Role modeling:** Using role modeling to exemplify the desired practice change.  Train-the-trainer: Training approach that turns employees into subject matter experts who can then teach other staff members.  **Feedback:** Soliciting stakeholder feedback and input about the practice change.  **Organizational incentives and rewards:** Positive reinforcement, including extrinsic incentives such as goal-sharing awards, performance reviews, promotions, and raises in salary, and less tangible incentives such as increased stature or respect. Also includes overall organizational incentives and rewards (i.e., benefits to the organization as a whole for adopting the change). | How did the new practice spread (either among individuals or among departments/systems)?  How did you get buy-in from stakeholders? |
|  | **Executing:** Carrying out or accomplishing the implementation according to plan.  Also use to capture statements related to making unplanned (or planned) changes to a practice change that has already been implemented. |  | How did the new practice spread (either among individuals or among departments/systems)?  How did you get buy-in from stakeholders? |
|  | **Reflecting and evaluating:** Quantitative and qualitative feedback about the progress and quality of implementation. |  | What were steps that were taken to guide the process (e.g., was it enforced by a certain group)? |
| **Outcomes:** The practical effects of the culture change.  *What are the effects of culture change?* | **Indicator:** Perceived indicators that informed participants that they were progressing toward or had achieved culture change. | **Quantitative: Quantitative measures (e.g., audits, quantitative data, ect.) that indicate progress towards a culture change.**  **Qualitative: Qualitative indicators (e.g., hearing about, witnessing, experiencing the change).** | How did you know when culture change was achieved?  Can you describe any of the indicators that let you or others know that you were progressing toward or had achieved culture change? |
|  | **Sustainment:** The degree to which the practice is sustained over time  *Only code a small chunk of data that best describes the example in a succinct and brief manner. | **Sustained change:** Examples of sustained practice changes. Code a practice change that remains in place, *even if it is modified after implementation*, to sustained change.  **Time-limited change:** Examples of time-limited practice changes. | Can you describe a specific example of culture change in your health system setting?  What is a specific example of culture change in any healthcare setting that you have heard of or that comes to mind?  Please describe a clinical practice change that was implemented and sustained for a substantial amount of time, even if ultimately ended.  Why do you think this new practice resulted in culture change and not only time-limited change (or vice-versa)? |
| **Double codes** | **+ :** The presence of a construct (e.g., presence of leadership engagement).  **-** : The absence of a construct (e.g., absence of leadership engagement).  **Illustrative quote**: Data that is especially rich in illustrating the respective code. |  |  |
