# Supplementary material for: Defining the transition from new to normal: a qualitative investigation of the clinical change process
Source: BMC Health Serv Res. 2024 Dec 18;24:1592. doi: 10.1186/s12913-024-12034-4 (PMC11653967; doi:10.1186/s12913-024-12034-4)
Supplement: Supplementary file 2 — Supplementary Material 2. [file 12913_2024_12034_MOESM2_ESM.docx]

**Culture Change in Clinical Practice: A Qualitative Investigation**

Introductory/Framing Script

We are conducting a study investigating what constitutes true “**culture change**” in clinical practice, meaning *how do new evidence-based practices, guidelines, or approaches move beyond initial uptake and later sustainment, and then become the “new normal”?* To give an example of what we mean, consider the common use of seatbelts today. When originally developed, seatbelts were novel and unknown. It’s likely that when seatbelts were first manufactured in cars, most people didn’t wear them, thought they were strange, or wore them incorrectly. Now, it is a near automatic reaction to sit in the car and reach for the seatbelt. Not only are seatbelts well-accepted by people and almost ubiquitously used, there are also laws and regulations that require the use of seatbelts. This example demonstrates the process of a new concept being introduced into a given community, and over time, becoming the norm.

Over the next 30 to 45 minutes, we will ask you briefly about your role and background, and then we would like to speak further with you about this process of evidence-based practice culture chance from your perspective. We are specifically interested in any relevant examples you can think of in your workplace.

Interview Questions

1. Please briefly tell me about your current role in quality improvement/implementation science.
2. Demographics:
3. **What is your gender?**

_1_ÿ Male

_2_ÿ Female

_3_ÿ Other:_________

1. **How old are you?**

_1_ÿ 26-35

_2_ÿ 36-45

_3_ÿ 46-55

_4_ÿ 56-65

_5_ ÿ >65

1. **Please indicate the degree(s) you have obtained. __________________**
2. **My primary job description is described by (select all that apply):**

_1_□ Educator (professor or similar)

_2_□ Clinician

_3_□ Researcher

_4_□ Leadership (e.g., manager, executive)

_5_□ Other:____________

1. **# of years in practice:** ______________
2. What does culture change in health care practice mean to you?
3. What do you see as the difference(s) between sustainability and culture change?
4. Can you describe a specific example of culture change in your health system setting?
   1. Branching options:
      1. *(Probe #1 if unable to think of an example)* What is a specific example of culture change in any healthcare setting that you have heard of or that comes to mind?
      2. *(Probe #2 if unable to answer)* Please describe a clinical practice change that was implemented and sustained for a substantial amount of time, even if ultimately ended.
   2. *(probe)* How did the idea for the new practice came about?
   3. *(probe)* Please describe the environment in which it was implemented.
   4. *(probe)* Who were the implementers?
   5. *(probe)* Why did the change in practice happen?
   6. *(probe)* How did the new practice spread (either among individuals or among departments/systems)?
   7. *(probe)* Who were the stakeholders?
   8. *(probe)* How did you get buy-in from stakeholders?
   9. *(probe)* What were steps that were taken to guide the process (e.g., was it enforced by a certain group)?
5. How did you know when culture change was achieved? *(SKIP if Probe #2 used in Question #3)*
   1. *(probe)* Can you describe any of the indicators that let you or others know that you were progressing toward or had achieved culture change?
   2. *(probe)* Why do you think this new practice resulted in culture change and not only time-limited change?
   3. *(ALTERNATE if Probe #2 used in Question #3)* Why do you think this new practice resulted in only time-limited change and not culture change?
6. Who else would you recommend to interview?

**PER IRB, do not ask for information that is not publicly available (for example, we will ask participants to provide names of nominees but will not ask for other information; we will look for contact information on university websites and similar).**
